# Total Hip Arthroplasty after Treatment of an Atypical Subtrochanteric Femoral Fracture in a Patient with Pycnodysostosis

**DOI:** 10.1155/2015/731910

**Published:** 2015-09-10

**Authors:** Takahito Yuasa, Koichi Maeda, Kazuo Kaneko, Kazunori Yoshikata

**Affiliations:** ^1^Department of Orthopaedic Surgery, Juntendo University, 2-1-1 Hongo, Bunkyo-ku, Tokyo 113-8421, Japan; ^2^Department of Orthopaedic Surgery, Juntendo University Nerima Hospital, 3-1-10 Takanodai, Nerima-ku, Tokyo 177-8521, Japan; ^3^Department of Orthopaedic Surgery, Yoshikata Hospital, 2-2-4 Nakamachi, Musashino, Tokyo 180-0066, Japan

## Abstract

The authors describe the case of a 51-year-old woman with an osteonecrosis of her right femoral head after treatment of an atypical subtrochanteric fracture caused by pycnodysostosis. She had this fracture after a low-trauma fall. She was of short stature with typical facial features, short stubby hands, and radiological features including open cranial sutures, obtuse mandible, and generalized skeletal sclerosis. The majority of cases of atypical subtrochanteric fractures are associated with long-term use of bisphosphonates; some occur in bisphosphonate-free patients. We report a rare case of total hip arthroplasty (THA) in a patient with pycnodysostosis who developed an osteonecrosis of the femoral head after treatment of an atypical subtrochanteric femoral fracture. We performed cementless THA in combination with a plate and cables. Cementless THA is a potential intervention in a patient with pycnodysostosis; although the bone quality may have been sclerotic, healing is not a problem in this condition.

## 1. Introduction

The authors describe a case of a 51-year-old woman with an osteonecrosis of her right femoral head after treatment of an atypical subtrochanteric fracture caused by pycnodysostosis. Pycnodysostosis is a rare and relatively benign osteosclerotic condition and was first described by Maroteaux and Lamy in 1962 [1]. It is a genetic disorder inherited as an autosomal recessive pattern. Genetically,* cathepsin K* gene mutations were found in patients with pycnodysostosis [2, 3]. Pycnodysostosis is characterized by short stature, with peculiar faces, consisting of bossing of the frontal bones and a shallow obtuse mandibular angle. Radiologically, the bones are sclerotic without loss of medullarization. Pycnodysostosis is a rare cause of an atypical subtrochanteric femoral fracture [4], and there are several reports of subtrochanteric femoral fracture in patients with this condition [5–7]. However, there is no report of an osteonecrosis in a patient with pycnodysostosis. Here we report a rare case of total hip arthroplasty in a patient with pycnodysostosis who developed osteonecrosis of the femoral head after treatment of an atypical subtrochanteric femoral fracture. The patient was informed that data concerning this case would be submitted for publication.

## 2. Case Report

A 51-year-old Japanese woman admitted to the hospital after a low-trauma fall. Clinical and radiological examination revealed an atypical subtrochanteric fracture of her right femur. She had a history of tibia fracture when she was twenty years old and treated by intramedullary nail. The patient was of short stature (138 cm) with typical facial features due to micrognathia, abnormal dentition, and a prominent nose. She had no abdominal organomegaly and denied consanguinity in her parents.

Laboratory investigations revealed no evidence of systemic, metabolic, or endocrine disorder. Her blood count and blood chemistry were normal. Other bone turnover markers, such as bone ALP and N-terminal telopeptide, were also normal.

**Figure 1:**
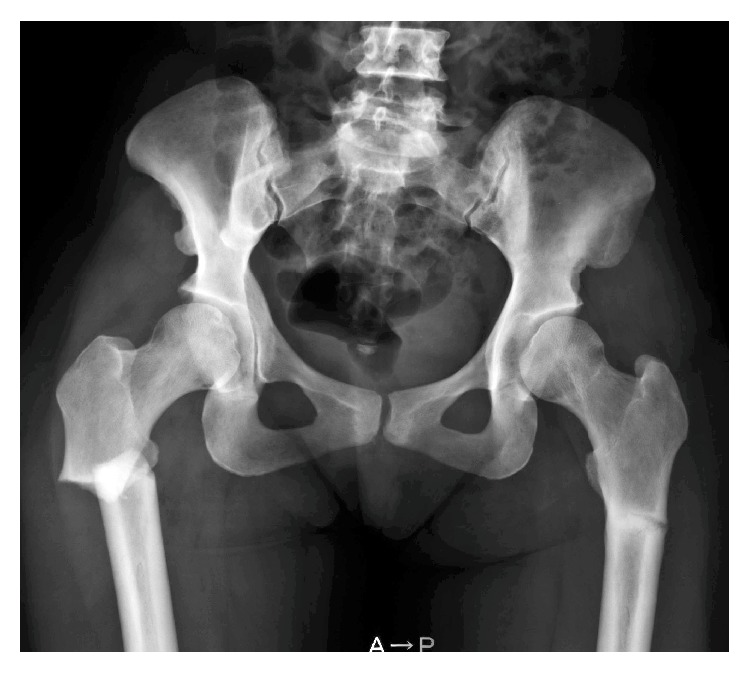
Radiograph showing subtrochanteric fracture of the right femur and a stress fracture in the left femur in the same region. Bones are sclerotic and medullary canal is visible.

**Figure 2:**
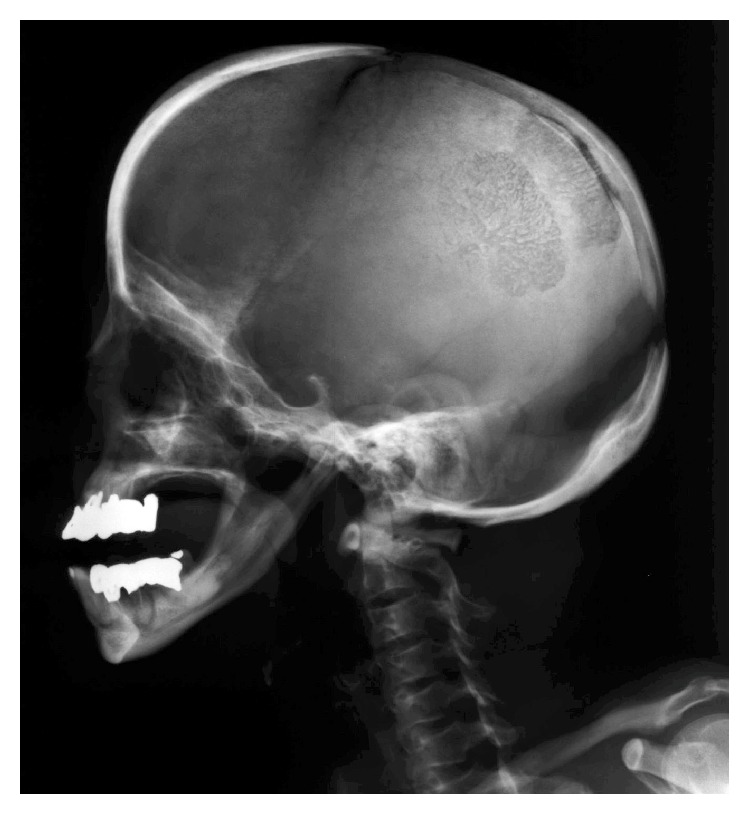
Lateral radiograph of the skull showed separated sutures, open fontanelle, and obtuse angle of mandible.

Radiographs of the right femur confirmed a noncomminuted, transverse subtrochanteric fracture with both lateral and medial cortical thickening, but without obliteration of the medullary canal, consistent with radiologic features of an atypical subtrochanteric femoral fracture [8] (Figure 1). Pelvic views demonstrated asymptomatic stress fracture in the left femur that involved the lateral cortex. A subsequent skeletal survey revealed cortical thickening of all long bones and an incompletely fused anterior fontanelle (Figure 2).

**Figure 3:**
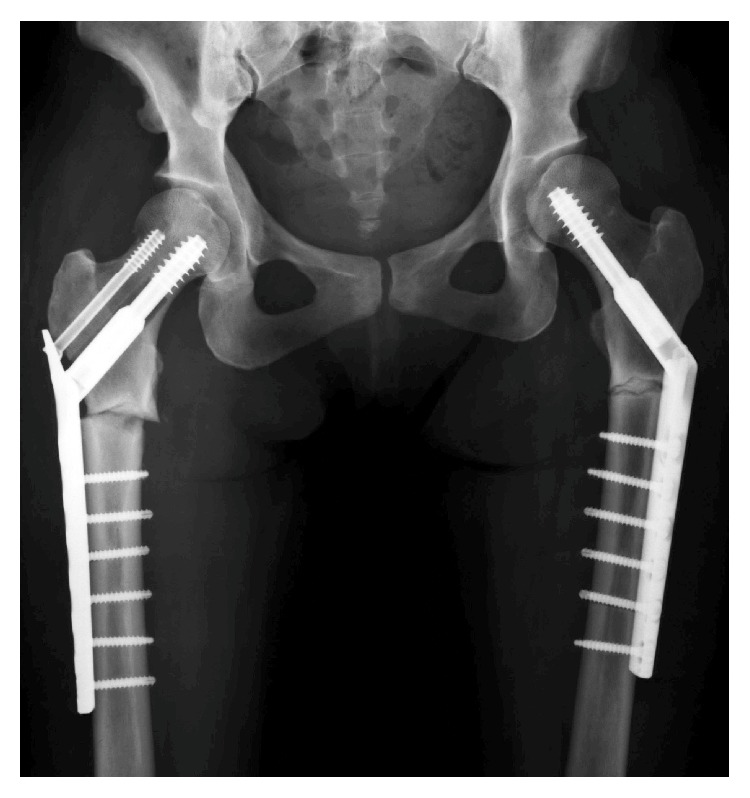
Radiograph after open reduction of the fractures with a plate and screws.

**Figure 4:**
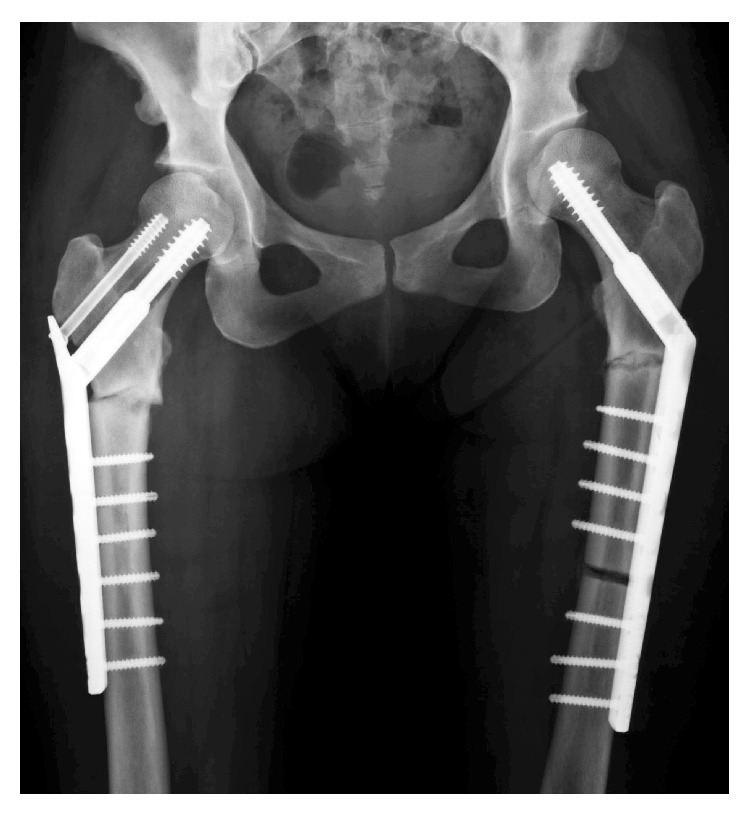
Radiograph after treatment of a fracture of the femoral shaft.

For the treatment of fracture, open reduction and internal fixation with a plate and screws were performed. Drilling, particularly in the femoral neck, was quite difficult as bone was sclerotic. For the opposite sided stress fracture, prophylactic operation, an internal fixation with a plate and screws, was performed (Figure 3). 10 days after operation of the left femur, fracture occurred at the distal screw and reoperation was performed using a long plate (Figure 4). Fracture healed well in 3 months and then the patient was able to bear full weight.

**Figure 5:**
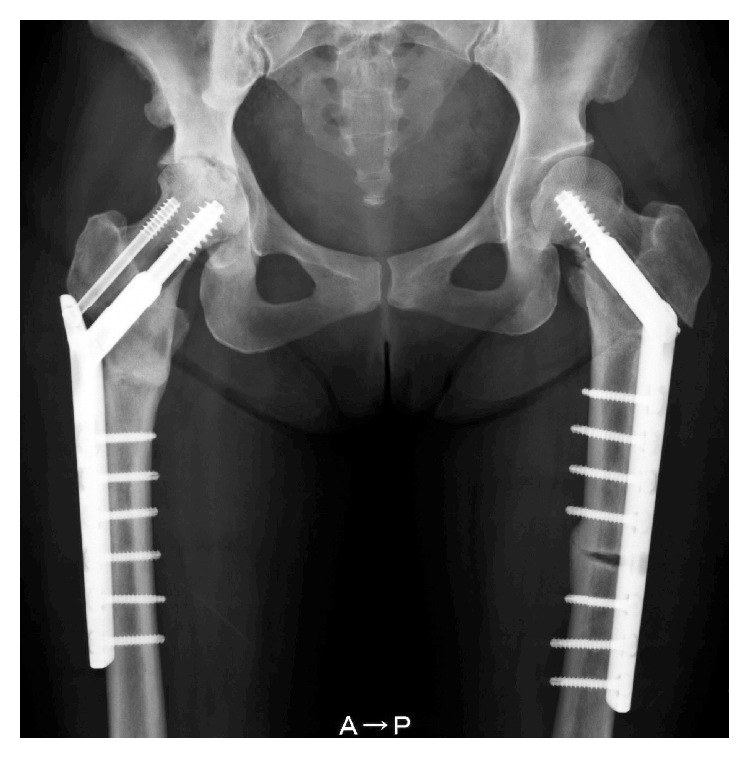
Radiograph showing advanced stage osteoarthritis of the right hip.

One year and 4 months after operation, she was suffering from right hip pain and could not walk. Plain radiological examination revealed narrowing of joint space of the right hip joint and osteonecrosis of the femoral head (Figure 5). The adaptation of total hip arthroplasty due to osteoarthritis secondary to severe pain, limited ambulatory ability, and restriction of activities of daily life was determined (Harris hip score, 37).

**Figure 6:**
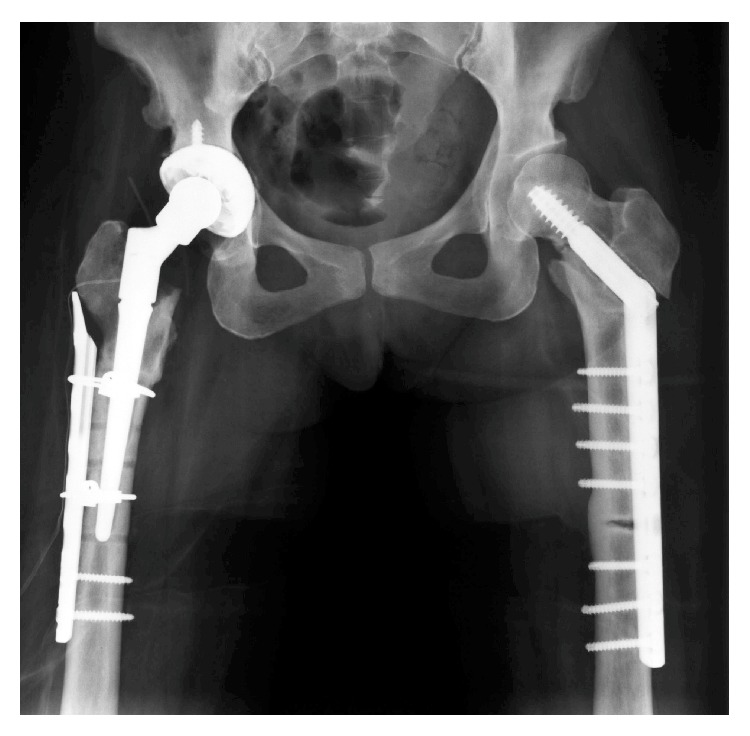
Radiograph after total hip arthroplasty with cable and plate fixation.

**Figure 7:**
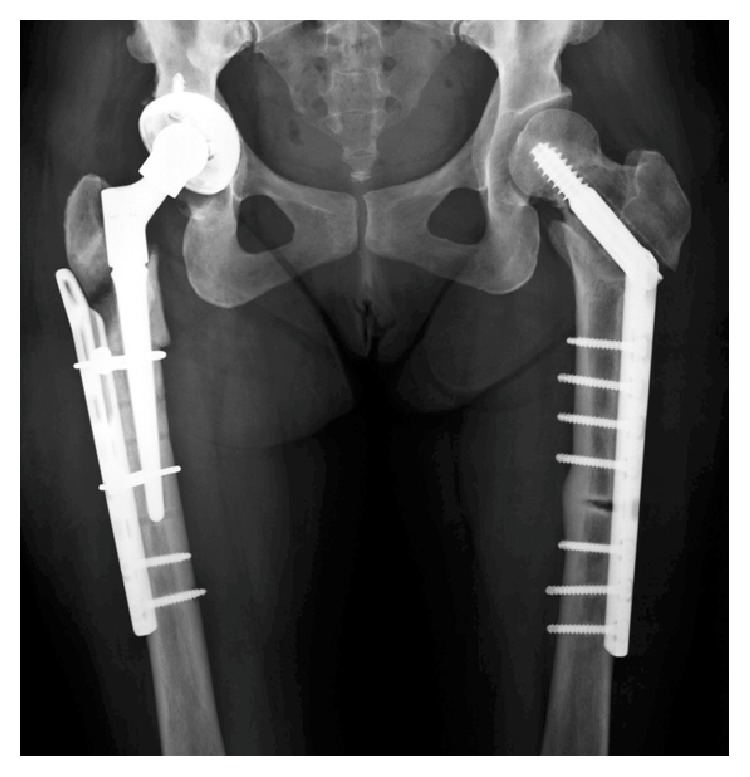
Radiograph of right hip one year after surgery, showing no migration of the implant.

The patient underwent cementless total hip arthroplasty using R3 acetabular system (Smith & Nephew, London, UK) and MODULUS stem with a modular neck (Lima Corporate, Udine, Italy) followed by fixation of the fracture with cable and plate system (Figure 6). This stem has the 5° finned stem taper which ensures optimal fixation across a wide range of femoral morphologies. Pathological examination of the femoral head was consistent with an osteonecrosis. Three weeks postoperatively, full-weight bearing was permitted and she could walk with a walking frame. At final follow-up, one year later, her symptoms had resolved and the patient had resumed normal activities (Harris hip score, 84). Radiographs showed no migration of implant (Figure 7).

## 3. Discussion

In a recent series of 1271 consecutive subtrochanteric fractures from Swedish registry, it was found that atypical fractures represent only 4.6% of the total [9]. The majority of cases of atypical subtrochanteric fractures are associated with long-term use of bisphosphonates (BPs); some occur in bisphosphonate-free patients [10], in whom genetic factors are likely to be important [4]. A diagnosis of the rare autosomal recessive osteosclerotic bone disease pycnodysostosis was made by clinical features. Pycnodysostosis is characterized by osteosclerosis, short stature, partial or total aplasia of the distal phalanges, bone fragility, clavicular dysplasia, and dysplasia of the skull. Osteoclasts are present in normal numbers but have abnormal cytoplasmic vacuoles containing bone collagen fibrils indicative of inadequate matrix degradation [11]. Genetically,* cathepsin K* gene mutations were found in patients with pycnodysostosis. This enzyme cleaves bone protein such as type I collagen, osteopontin, and osteonectin. The localization and mutation of cathepsin K in activated osteoclasts have been characterized [12]. This enzyme is secreted into the subosteoclastic space where bone matrix is degraded [13].

The treatment of this disease is restricted to symptomatic management of fractures and other skeletal problems. The operative treatment for the patients with fracture is challenging for the orthopaedic surgeons because of the unusual problems imposed by the hard-but-brittle bone characteristics of the disease. The patient with this syndrome tends to suffer fractures as a result of mild trauma because of the abnormal composition of the bone, indicated by its increased density. Atypical subtrochanteric fracture is frequent in pycnodysostosis as biomechanically the bones are more resistant to compression than to slow tension, particularly in that anatomic area [5].

There are no similar reports in the literature describing the osteonecrosis of the femoral head after treatment of an atypical subtrochanteric femoral fracture in a patient with pycnodysostosis. In this disease, reaming, particularly in the diaphyseal area, may be quite difficult as medullary canal is almost blocked although it was radiologically visible [12]. In this case, reaming of the femoral neck was quite difficult and it took longer time at the treatment of the fracture. We speculate that the heat stress by reaming is caused the osteonecrosis of the femoral head. For the treatment, we performed the cementless total hip arthroplasty. We attempted to gain biological fixation of the implant; therefore, we selected a cementless fixation. Although the bone quality may have been sclerotic, healing is not a problem in patients with pycnodysostosis.

## References

[1] Maroteaux P., Lamy M. (1962). La pycnodysostose. *La Presse Médicale*.

[2] Gelb B. D., Edelson J. G., Desnick R. J. (1995). Linkage of pycnodysostosis to chromosome 1q21 by homozygosity mapping. *Nature Genetics*.

[3] Gelb B. D., Shi G.-P., Chapman H. A., Desnick R. J. (1996). Pycnodysostosis, a lysosomal disease caused by cathepsin K deficiency. *Science*.

[4] Yates C. J., Bartlett M. J., Ebeling P. R. (2011). An atypical subtrochanteric femoral fracture from pycnodysostosis: a lesson from nature. *Journal of Bone and Mineral Research*.

[5] Roth V. G. (1976). Pycnodysostosis presenting with bilateral subtrachanteric fractures: case report. *Clinical Orthopaedics and Related Research*.

[6] Kundu Z. S., Marya K. M., Devgan A., Yadav V., Rohilla S. (2004). Subtrochanteric fracture managed by intramedullary nail in a patient with pycnodysostosis. *Joint Bone Spine*.

[7] Bor N., Rubin G., Rozen N. (2011). Fracture management in pycnodysostosis: 27 years of follow-up. *Journal of Pediatric Orthopaedics B*.

[8] Shane E., Burr D., Ebeling P. R. (2010). Atypical subtrochanteric and diaphyseal femoral fractures: report of a task force of the American Society for Bone and Mineral Research. *Journal of Bone and Mineral Research*.

[9] Schilcher J., Michaëlsson K., Aspenberg P. (2011). Bisphosphonate use and atypical fractures of the femoral shaft. *The New England Journal of Medicine*.

[10] Tan S. C., Koh S. B. J., Goh S. K., Howe T. S. (2011). Atypical femoral stress fractures in bisphosphonate-free patients. *Osteoporosis International*.

[11] Everts V., Aronson D. C., Beertsen W. (1985). Phagocytosis of bone collagen by osteoclasts in two cases of pycnodysostosis. *Calcified Tissue International*.

[12] Yamashita D. S., Dodds R. A. (2000). Cathepsin K and the design of inhibitors of cathepsin K. *Current Pharmaceutical Design*.

[13] Nishi Y., Atley L., Eyre D. E. (1999). Determination of bone markers in pycnodysostosis: effects of cathepsin K deficiency on bone matrix degradation. *Journal of Bone and Mineral Research*.

